# A Comparative Pilot Study on the Treatment of Uterine Fibroids Using Traditional Medicines and Intrauterine Enema Therapy

**DOI:** 10.7759/cureus.91387

**Published:** 2025-09-01

**Authors:** Shikha Singh, Kavita Saini, Neha Garg

**Affiliations:** 1 Department of Gynecology, Institute of Medical Sciences, Banaras Hindu University, Varanasi, IND; 2 Department of Medicinal Chemistry, Institute of Medical Sciences, Banaras Hindu University, Varanasi, IND

**Keywords:** ayurveda, enema, fibroid, traditional medicine, uterine fibroid

## Abstract

Background and objectives: Uterine fibroids (UFs) are the most typical benign tumors of the uterus of women of childbearing age, especially nulliparous women. Symptomatic UFs are associated with heavy menstrual bleeding, pelvic pain, infertility, etc. The study aims to evaluate the efficacy of traditional medicines and enema therapy in reducing fibroid size and associated symptoms.

Methods: In this pilot study, 22 patients from Varanasi, Uttar Pradesh, diagnosed with UFs were divided into two groups: the trial (n=11) and control (n=11) groups. Trial group patients received Ayurvedic medicines along with *Uttar Basti *(enema therapy) using *Kapharbuda-har *oil (prepared in the Ayurvedic pharmacy of Banaras Hindu University, UP, India) for three months. On the other hand, control group patients were given Ayurvedic medicines along with *Uttar Basti *using *til *(sesame) oil over the same period. After the treatment, the fibroid size and associated symptoms were monitored.

Results: After three months of treatment, all patients in the trial group reported symptomatic relief. Complete fibroid resolution was observed in two patients, while one fibroid completely resolved in another patient who had three fibroids. Five patients showed a reduction in fibroid size, whereas one patient experienced an increase in size. On the other hand, the control group patients didn’t get relief from symptoms, and the fibroid size either increased or showed no significant change.

Conclusion: From the present study, we can conclude that Ayurvedic treatments could be effective in controlling the size and symptoms associated with UFs.

## Introduction

Uterine fibroids (UFs) are monoclonal tumors of the uterus of reproductive-age females [1]. About 70-80% of females suffer from fibroids during their lifetime [2]. But many females remain asymptomatic; around 25% of reproductive-age females experience symptoms of fibroids [3]. These tumors can lead to a range of issues, such as irregular uterine bleeding, pelvic pain, bladder or bowel-associated problems, and repeated pregnancy loss. Each year, over 200,000 hysterectomies are carried out in the US due to these tumors [4].

UFs are monoclonal and develop from a fibroid stem cell that formed due to a genetic mutation in myometrial stem cells. Further proliferation of fibroid stem cells with higher extracellular matrix production leads to fibroid formation [4,5]. UFs modify the environment and shape of the uterus, thus hampering sperm transport and implantation of embryos, leading to reduced fertility. The presence of fibroids during pregnancy increases the chances of abortion, placental previa, preterm birth, placental abruption, cesarean delivery, intrauterine fetal death, preeclampsia, preterm premature rupture of membranes (PPH), and breech presentation [5,6]. UFs are hormone-dependent tumors; estrogen and progesterone are reported to promote the growth of fibroids [4]. Fibroid growth regresses in menopausal women [2]. Four types of genetic alterations are reported in UFs: mediator complex subunit 12 (MED 12) mutation, which is a missense mutation; higher expression of high mobility group AT-hook 2 (HMGA2), which is a chromosomal rearrangement; deficiency of fumarate hydratase; and deletion of collagen type 4-α-5 chains (COL4A5) and collagen type 4-α-6 chains (COL4A6) [7,8]. The frequency of MED12 mutations, HMGA2 mutations, FH deficiency, and COL4A5 and COL4A6 deletions is 50-80%, 10-20%, 1-2% and less than 1%, respectively [8]. Currently, effective treatment options are surgery-based. Several modern medicines like Ullipristal are used for the treatment of UFs, but they are associated with side effects [1]. So there is a need for a non-surgical method for the treatment of UFs that can alleviate symptoms while preserving fertility.

Ayurveda is an ancient Indian system of medicine that plays a key role in the treatment of various gynecological diseases [9]. Ayurvedic medicines, along with *Uttar Basti *(enema therapy), have been found effective in managing size and symptoms associated with UF. A case report of a 45-year-old female with symptomatic UF showed significant size reduction after three months of *Kshar Kasisadi* oil *Uttar Basti* [10]. Similarly, three months of *Uttar Basti* with *Palash Kshara Taila*, *Virechna* *Basti *(controlled medicated purgation enema therapy), and *Lekhna Basti *(scraping/reducing enema therapy) reduced fibroid size and improved symptoms [11]. In Ayurveda, UFs are associated with *garbhasya arbuda,* a type of tumor/growth [12]. In the present pilot study, UFs are treated with Ayurvedic medicine along with intrauterine *Uttar Basti* with *Kapharbuda-har *oil (prepared in the Ayurvedic pharmacy of Banaras Hindu University, UP, India). Intrauterine *Uttar Basti* is a type of enema therapy in which a medicated oil is inserted into the uterus of a female, and it works by balancing the *vata-dosha *(Ayurvedic mind-body element associated with air and space), cleaning the urinogenital tract, and nourishing tissues. It is used to treat various gynecological issues [13].

## Materials and methods

Sample size calculation

The sample size has been calculated by using standard parameters (α = 0.05, power = 80%) for a meaningful difference in fibroid size, by the given formula:

\(n = \frac{(Z_{\alpha/2} + Z_{\beta})^2 \times 2}{\left(\frac{\Delta}{\sigma}\right)^2}\)

Based on the assumption of a moderate to large effect size, the minimum sample required was estimated to be nine patients per group. Assuming a 15% dropout rate, the final required sample sizes were 11 patients per group. All the patients were randomly divided into a control and a trial group using a chit-based method.

Study parameters

Parameters considered were intervals between two menstrual cycles, duration of menstrual bleeding, and amount of menstrual bleeding per day, measured by sanitary pads used per day. Pelvic pain during menstruation and the size of UFs.

Outcomes

The primary outcome was improvement in the symptoms associated with UFs and in the quality of life in patients with UFs after the treatment. The secondary outcome was the reduction or complete resolution of UFs after treatment, as indicated by the USG report.

Trial design

The study was approved by the Institutional Ethics Committee of the Institute of Medical Science, Banaras Hindu University, U.P., (Dean/2020/EC/2147) and registered with the Clinical Trials Registry (CTRI) under the registration number CTRI/2022/05/042372, which was updated every six months. Patients were enrolled in the OPD of Prasuti Tantra, Banaras Hindu University. The patients were suffering from UFs as per the USG report. Written informed consent was obtained from the patients for the study. All the patients were divided into two groups, a trial group (n=11) and a control group (n=11), but two patients in both groups didn’t complete the treatment, so they were excluded from the study. Both groups were prescribed medication continuously for three months, which included *Godanti Bhasm* (Dhootapapeshwar, Panvel, India), *Pushyanug Churna* (Patanjali, Divya Pharmacy, Haridwar, India), *Bol Parpati *(Ashtang, Mehsana, India), *Lodhrasava* (DAV Pharmacy, Jalandhar, India), and an iron supplement. The quantity of the dose with the prescription is given in Table 1. Patients in the trial group received intrauterine *Uttar Basti* with *Kapharbuda-har* oil for seven consecutive days following the end of their menstrual periods for three months. Similarly, patients in the control group were given intrauterine *Uttar Basti* with *til* (sesame) oil for the same duration after their menstruation had ended. The timelines of the *Uttar Basti *with the last menstruation are given in Table 2 (trial group) and Table 3 (control group). After three months of treatment, patients underwent pelvic USG, and symptoms associated with UF were also monitored.

**Table 1:** Medications prescribed to the patients.

Sr. No.	Drug name	Chemical composition	Dose quantity	Prescribed dose	References
1.	*Godanti Bhasm *(Gypsum, Dhootapapeshwar)	Calcium sulphate dihydrate	500 mg	2 times/day	[14,15]
2.	*Pushyanug Churna *(Patanjali, Divya Pharmacy)	*Patha (Cissampelos pareira), Jamun (Syzygium cumini), Aam (Mangifera indica), Pasanbhed (Bergenia ligulata), Rasaut (Extractum berberis), Ambashtha (Tamarix articulata), Mochras (Salmalia malabarica), Manjistha (Rubia cordifolia), Kamalkeshar (Nelumbo nucifera), Nagkeshar (Mesua ferrea), Atis (Aconitum heterophylllum), Nagarmotha (Cyperus rotundus), Bel (Aegle marmelos), Lodhra (Symplocos racemosa), Geru, Kayphal (Myrica nagi), Kalimirch (Piper nigrum), Sonth (Zingiber officinale), Munkka (Vitis vinifera), Lal chandan (Pterocarpus santalinus), Shyonak (Oroxylum indicum), Indrayava (Holarrhena antidysenterica), Anantamul (Hemidesmus indicus), Dhai (Woodfordia fruticosa), Mulethi (Glycyrrhiza glabra), Arjun (Terminalia arjuna), Geru*: 3.84 g each of the ingredients	1 g	2 times/day	[16,17]
3.	*Bol Parpati *(Ashtang)	*Bola (Commiphora myrrha*): 20 g, *Shudha Gandhaka* (herbal purified sulphur): 10 g, *Shudha Parada *(herbal purified mercury): 10g	250 mg	2 times/day	[18,19]
4.	*Lodhrasava* (DAV Pharmacy)	*Lodhra (Symplocos racemosa), Pushkarmool (Inula racemosa), Murva (Marsdenia tenacissima), Kachur (Curcuma zeodaria), Brihataila (Amomum subulatum), Vidang (Embelia ribes), Bahera (Terminalia bellerica), Ajwain (Trachyspermum ammi), Harar (Terminalia chebula), Amla (Emblica officinalis), Chavya (Piper retrofractum), Supari (Acacia catechu), Priyangu (Callicarpa macrophylla), Inderayanmool (Citrillus colocynthis), Kutaki (Picrorrhiza kurroa), Tagar (Valeriana wallichii), Chirayata (Swertia chirayita), Bharangi (Clerodendrum serratum), Chitrakmool (Plumbago zeylanicum), Kuth (Saussurea lappa), Patha (Cissampelos pareira), Pipplamool (Piper longum root), Ativisha (Aconitum hetrophyllum), Inderyav (Citrullus colocynthis), Tejpatra (Cinnamum inners), Nagkeshar (Mesua ferrea), Mustak (Cyperus rotundus), Dalchini (Cinamum zeylanicum)*: 1 part each of the above mentioned ingredients, *Dhataki (Woodfordia fruticosa*): 20 parts, water, *Guda* (Jaggary): 130 parts	20 ml	2 times/day	[16,20,21]
5.	Iron	Ferrous sulfate	1 tablet	1 time/day	[20]

**Table 2:** Timelines of Uttar Basti patients (trial group), with last menstruation.

Details	Last menstruation before 1st *Uttar Basti*	1^st^ cycle of *Uttar Basti*	Last menstruation before 2nd *Uttar Basti*	2^nd^ cycle of *Uttar Basti*	Last menstruation before 3rd *Uttar Basti*	3^rd^ cycle of *Uttar Basti*
Case 1	01.04.2024	10.04.2024	27.04.2024	02.05.2024	26.05.2024	04.06.2024
Case 2	12.06.2022	20.06.2022	10.07.2022	18.07.2022	17.08.2022	25.08.2022
Case 3	12.08.2023	20.08.2023	15.09.2023	21.09.2023	13.10.2023	20.10.2023
Case 4	12.02.2022	20.02.2022	11.03.2022	19.03.2022	18.04.2022	23.04.2022
Case 5	24.03.2023	30.03.2023	29.04.2023	05.05.2023	03.05.2023	10.06.2023
Case 6	15.05.2023	21.05.2023	17.06.2023	23.06.2023	22.07.2023	29.07.2023
Case 7	12.10.2023	20.10.2023	15.11.2023	22.11.2023	18.12.2023	25.12.2023
Case 8	25.07.2023	01.08.2023	21.08.2023	28.08.2023	20.09.2023	27.09.2023
Case 9	01.02.2023	08.02.2023	04.03.2023	11.03.2023	10.04.2023	17.04.2023

**Table 3:** Timelines of Uttar Basti patients (control group), with last menstruation.

Details	Last menstruation before 1st *Uttar Basti*	1^st^ cycle of *Uttar Basti*	Last menstruation before 2nd *Uttar Basti*	2^nd^ cycle of *Uttar Basti*	Last menstruation before 3rd *Uttar Basti*	3^rd^ cycle of* Uttar Basti*
Case 1	05.04.2022	10.04.2022	04.05.2022	10.05.2022	25.06.2022	03.07.2022
Case 2	01.05.2022	10.05.2022	05.06.2022	12.06.2022	07.07.2022	11.07.2022
Case 3	25.02.2022	10.06.2022	03.07.2022	12.07.2022	01.08.2022	06.08.2022
Case 4	05.11.2022	13.11.2022	05.12.2022	15.12.2022	20.01.2023	28.01.2023
Case 5	15.10.2022	20.10.2022	15.11.2022	19.11.2022	17.12.2022	24.12.2022
Case 6	04.09.2022	12.09.2022	14.10.2022	22.10.2022	25.11.2022	03.11.2022
Case 7	20.11.2022	28.11.2022	23.12.2022	28.12.2022	31.01.2023	05.02.2023
Case 8	10.01.2023	19.01.2023	07.02.2023	14.02.2023	08.03.2023	13.03.2023
Case 9	06.12.2021	12.12.2021	10.01.2022	14.01.2022	12.02.2022	17.02.2022

Inclusion and exclusion criteria

Married females of premenopausal age diagnosed with UFs who were willing to participate were included in the study. All the females included in the study were suffering from symptomatic fibroids. All the patients had normal respiratory rate, cardiovascular activity, and nervous systems. All patients gave written consent for the study and had not undergone any hormonal therapy for at least three months prior to the study. The study excluded unmarried, menopausal, and cancer-afflicted females.

Patient history

The patients enrolled in the study belonged to Varanasi city of Uttar Pradesh, located in Northern India. The history of the patients, including age, symptoms associated with UFs, and fibroid size, is provided in Tables 4, 5 for the trial and control groups, respectively.

**Table 4:** History of patients of the trial group.

Details	Age	Symptoms	Location and type of fibroids	Fibroid size
Case 1	25 years	Abdominal pain during menses, low abdominal pain, and heavy menstrual bleeding for the last two years	Posterior myometrium of the uterus, intramural fibroid	14.5X19.8 mm
Case 2	33 years	Heavy menstrual bleeding and menses between periods	Anterior, posterior, and posterior myometrium of the uterus, respectively, intramural fibroids	4.9X5.9 mm, 5.1X4.4 mm, and 5.2X4.67 mm
Case 3	24 years	Menses between the periods, abdominal pain, and infertility	Anterior myometrium of the uterus, intramural fibroid	10.6X8.7 mm
Case 4	41 years	Heavy menstrual bleeding, abdominal pain during menses, and intermenstrual bleeding	Anterior myometrium of the uterus, submucosal fibroid	16.1X13.8 mm
Case 5	29 years	Excessive menstrual bleeding and abdominal pain during menstruation	Posterior myometrium of the uterus, intramural fibroids	7.5X4.5 mm
Case 6	35 years	Heavy menstrual bleeding and irregular menses	Anterior wall of the uterus, subserosal fibroid	41.5X31.6 mm
Case 7	42 years	Heavy menstrual bleeding and pain during menses	Left fundo lateral wall, intramural fibroid	60X52 mm
Case 8	35 years	Heavy menstrual bleeding, pain during menses, and passage of blood clots during menstruation	Posterior myometrium of the uterus, intramural fibroid	12.7X9.1 mm
Case 9	32 years	Heavy menstrual bleeding and intermenstrual bleeding	Anterior myometrium of the uterus, intramural fibroid	5.5X60 mm

**Table 5:** History of patients of the control group.

Details	Age	Symptoms	Location and type of fibroids	Fibroid size
Case 1	42 years	Irregular menses, heavy menstrual bleeding, and reduced interval between 2 cycles	Posterior myometrium of the uterus, intramural fibroids	19X14 mm
Case 2	18 years	Abdominal pain and heavy menstrual bleeding during menses	Anterior myometrium of the uterus, intramural fibroid	29X30 mm
Case 3	45 years	Menses between the periods, abdominal pain	Anterior wall, posterior wall, intramural fibroid	46X35 mm, and 46X43 mm
Case 4	36 years	Heavy menstrual bleeding and irregular menses	Anterior wall of uterus, intramural left lateral wall of the uterus, subserosal fibroid	42.2X46.3 mm, 35.7X30.7 mm
Case 5	40 years	Heavy menstrual bleeding during menses, low back pain, and weakness	Anterior wall of the uterus, intramural fibroid	48.3X46.1 mm
Case 6	40 years	Heavy menstrual bleeding and irregular menses	Posterior myometrium of the uterus, intramural fibroids	43.1X33.2 mm
Case 7	40 years	Abdominal pain and heavy menstrual bleeding during menses	Posterior myometrium of the uterus, intramural fibroids	14.4X12.2 mm
Case 8	35 years	Heavy menstrual bleeding and pain during menses	Fundal wall, subserosal posterior wall, subserosal posterior wall, intramural	76X62 mm, 54X49 mm, and 43X36 mm
Case 9	22 years	Heavy menstrual bleeding and irregular menses	Posterior wall, intramural	13X13 mm

Data analysis

Data were analyzed using Microsoft Excel (Microsoft Corporation, Redmond, Washington, United States). Data significance in both groups was evaluated using Student’s paired t-test at p < 0.05.

## Results

Reduction of fibroid size

**Figure 1:**
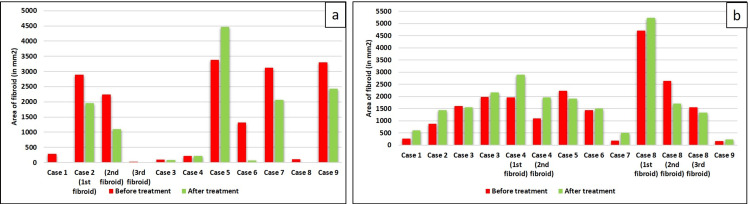
Reduction in the size of fibroids. a: comparison of the size of the fibroid (in mm²) before treatment and after treatment (trial group); b: comparison of the size of the fibroid (in mm²) before treatment and after treatment (control group). mm: millimeter

After three months of treatment, the trial group patients’ investigations revealed a significant decrease in fibroid size (p=0.03). As per the USG report, in two patients, fibroids (intramural and subserosal) resolved completely, and in one patient, one fibroid (intramural) completely resolved. In other patients, the fibroid size was reduced in comparison to before treatment. In one patient, the fibroid (intramural) size increased. Figure 1a and Table 6 represent the size of the fibroid before and after the treatment. On the other hand, in the control group, fibroid size either increased or remained the same (p=0.11), as represented in Figure 1b and Table 6.

**Table 6:** Size of fibroids in the trial and control groups.

Details	Trial group		Details	Control group	
	Area of fibroid before treatment	Area of fibroid after treatment		Area of fibroid before treatment	Area of fibroid after treatment
Case 1	287.1 mm^2^	0 mm^2^	Case 1	266 mm^2^	600 mm^2^
Case 2	2891 mm^2^, 2244 mm^2^, 24.284mm^2^	1095.86 mm^2^, 1095.99 mm^2^, 0 mm^2^	Case 2	870 mm^2^	1440 mm^2^
Case 3	92.22 mm^2^	79.8 mm^2^	Case 3	1610 mm^2^, 1978mm^2^	1550 mm^2^, 2166 mm^2^
Case 4	222.18 mm^2^	220.8 mm^2^	Case 4	1953.86 mm^2^, 1095.99 mm^2^	2896.4 mm^2^, 1966.07 mm^2^
Case 5	3375 mm^2^	4466 mm^2^	Case 5	2226.63 mm^2^	1911 mm^2^
Case 6	1311.4 mm^2^	69.12 mm^2^	Case 6	1430.92 mm^2^	1505 mm^2^
Case 7	3120 mm^2^	2064 mm^2^	Case 7	175.68 mm^2^	509.95 mm^2^
Case 8	115.57 mm^2^	0 mm^2^	Case 8	4712 mm^2^, 2646 mm^2^, 1548mm^2^	5229 mm^2^, 1710mm^2^, 1332mm^2^
Case 9	3300 mm^2^	2430 mm^2^	Case 9	169 mm^2^	234 mm^2^

Improvement of symptoms

**Figure 2:**
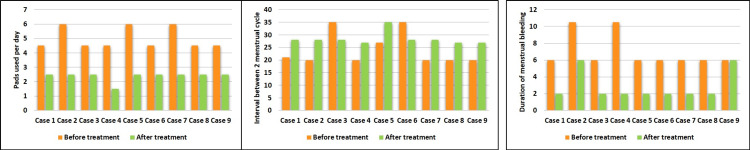
Comparison of symptoms associated with uterine fibroids before and after treatment. a: comparison of sanitary pads used per day by patients in the trial group before treatment and after treatment; b: comparison of the interval between two menstrual cycles (in days) in the patients of the trial group before treatment and after treatment; c: comparison of duration of menstrual bleeding (in days) before treatment and after treatment of the trial group. mm: millimeter

After three months of treatment, in the trial group, improvement in symptoms associated with UFs was observed. Menstrual bleeding was normal after the treatment, indicated by pads used per day (p <0.001), as shown in Figure 2a. A regular menstruation cycle of 28-30 days was observed after the treatment in the trial group (p<0.001), which was of around 20 days before the treatment, Figure 2b. Also, the duration of menstrual bleeding was reduced after the treatment (p <0.001), Figure 2c. On the other hand, in the control group, no improvement in the symptoms associated with UFs was observed.

## Discussion

*Uttar Basti* is a P*anchakarma* procedure (five-fold detoxification and rejuvenation treatment) that plays a significant role in the management of various gynecological disorders, including tubal blockage, cervical erosion, vaginal mucosal defects, endometriosis, and infertility etc. [22,23]. In intrauterine *Uttar Basti,* the drug is administered through the vaginal route during the morning time after the cessation of menstruation, because at that time vaginal and uterine openings are wide to absorb the administered drug readily [23]. It works by balancing *vata-dosha*, cleaning the urinogenital tract, nourishing tissue, stimulating various pathways of neuro-endocrine signaling [13], and targeting issues like tubal blockages [24] or urethral strictures [25] with specific medicated oils. It boosts blood circulation and improves organ function, showing effectiveness in managing infertility and related conditions [26,27].

In several clinical studies, *Uttar Basti *and Ayurvedic medications are also reported to reduce fibroid size and associated symptoms [10,11]. In a case study, a female patient of age 45 years, diagnosed with UF, was treated with *Kshar Kasisadi* oil for three menstrual cycles. After three months of treatment, a significant reduction in fibroid size was observed in the patient, as per the USG report [10]. A similar study also shows the same result, in which a patient with UFs along with irregular uterine bleeding between the menstrual cycles was treated with *Uttar Basti* with *Palash Kshara Taila, Virechna Basti*, and *Lekhna Basti* for three months. After the treatment, a reduction in fibroid size was observed along with improvement in symptoms associated with UFs [11]. In a case series, three patients diagnosed with uterine disorders, including UFs and adenomyosis, were treated with *Panchakarma* therapy, *Virechna*, which was then followed by *Uttar Basti *as well as shaman therapy. After five months of treatment, the USG report indicated the absence of uterine disorders, UFs, and adenomyosis [28].

In Ayurveda, we can correlate symptomatic UFs (especially intramural fibroids) with *kaphaja arbuda*, which is a type of tumor. *Kapharbuda-har lepa*, which is formed of Ayurvedic drugs, *Langali (Gloriosa superba*), *Himsra (Capparis spinosa*), and *Granthiparni (Leonotis nepetifolia*): one part each; *Tamara bhasma (Copper ash*): ½ part, *Apamarga kshar-jala* (alkaline water): ½ part of *Apamarga kshar* with water, eight times of Ayurvedic drugs, and *Goumuta* (cow urine): eight times of Ayurvedic drugs, is described for the treatment of *arbuda* [29]. As we can’t apply *lepa (paste)* directly to UF, *Kapharbuda-har* oil was prepared by using the above-mentioned ingredients, and *til* oil with four times the amount of Ayurvedic drugs was used as a base. These drugs act by being absorbed into the tissue of the uterus. These drugs have various applications in gynecological problems. Other drugs were prescribed to reduce the symptoms associated with UF [12].

In the trial group, pads used by the patients were five to six in number per day before the treatment, which reduced to two to three per day after the treatment, along with a reduction in the duration of menstrual bleeding. The menstrual cycle, which was 20-35 days before the treatment, became normal (28-29 days) after the treatment. A reduction in the number of pads used after treatment indicates a decrease in menstrual bleeding and a reduction in the size of fibroids (confirmed by the USG report) after the treatment, suggesting the efficacy of the treatment. Also, in one patient, the fibroid size increased after treatment, but symptoms associated with UF improved, which was the primary outcome of the study. Also, no potential risks associated with medicated *Uttar Basti *were observed in either group. Therefore, we can say that all patients in the trial group achieved the primary outcomes of the study, marking a significant milestone in its success. So, based on the results of the study, we can speculate that Ayurvedic treatment options are effective in the treatment of UFs, without any side effects. Fibroid size didn’t increase in the patients after a few months of completion of treatment.

UFs are hormone-dependent tumors; estrogen and progesterone are responsible for the pathogenesis of UFs. The medicated oil might work by modulating or reducing the expression of estrogen and progesterone receptors, resulting in disruption of the pathogenesis of UFs. However, further experimental evaluations are required to determine the mechanism of action of the given medications.

Limitations

The restricted sample size is the one limitation of the study. Several patients didn’t follow up after the treatment, as they were relieved from the symptoms associated with UFs. Secondly, we did not have a standard group that should have been administered modern drugs. So we would not be able to compare the current treatment with modern medicines of UF.

## Conclusions

Based on the results of the present study, we can speculate on the effectiveness of Ayurvedic therapies for the treatment of UFs. Ayurvedic drugs, as well as *Uttar Basti* of medicated oil, are both effective in reducing the size of fibroids and in addressing the symptoms associated with UFs. Ayurvedic drugs and *Uttar Basti* might show a synergistic effect. However, additional experimental evaluations are required to determine the mechanism of action of drugs, as well as *Uttar Basti.*
